# Estimation of the Rural Dog Population Within a Mega-City: An Example in Jiading District, Shanghai

**DOI:** 10.3389/fvets.2021.630180

**Published:** 2021-07-05

**Authors:** Xiujuan Wu, Viola Yifei Yu, Zhong Huang, Jun Lu, Wenhong Tang, Sufang Shen, Luming Xia, Jiuchao Zhu, Jian Wang, Jiansheng Chen, Guanming Chen, Yi Bian, Michael P. Ward, Hongjin Zhao

**Affiliations:** ^1^Shanghai Animal Disease Control Center, Shanghai, China; ^2^Faculty of Science, Sydney School of Veterinary Science, The University of Sydney, Sydney, NSW, Australia; ^3^Jiading District Animal Disease Control Center, Shanghai, China

**Keywords:** dog, population, Shanghai, rural, enumeration

## Abstract

Rural dog populations have long been recognized to be inadequately managed in terms of disease control and prevention. In this study we consider dog management in rural Shanghai and its implications for rabies control in the entire metropolitan area of Shanghai. The prerequisite to improve rabies vaccination coverage in rural Shanghai depends on a proper enumeration of the total rural dog population. In this study we selected one of the nine administrative districts in Shanghai (Jiading), within which there are 7 towns and 2 industrial zones (township-level division) that contain agricultural areas. A total of 9 villages (rabies model villages) were chosen from each township-level division in Jiading, and an additional 3 non-model villages were also included in the study. A household questionnaire survey was implemented in all 12 villages recruited. In 3 of the model villages and the 3 non-model villages chosen as a comparison, two methods of enumeration—a sight-resight survey and a household census survey—were implemented. Results from the household survey in these 6 villages showed that among the total 1,560 owned dogs, 80.4% were Chinese Garden Dogs, 69.1% were aged 1 to 3 years, 49.2% were homebred, and 88.3% were kept for the purpose of guarding the house. However, only 3.7% of the owned dogs were desexed. There was a higher proportion of chained or confined dogs in model compared to non-model villages. The model villages had an absolute rabies vaccination coverage of 100% among its owned dog population and a smaller number of stray dogs. It was also identified that the two enumeration methods yielded similar counts (*P* = 0.12), particularly within smaller villages. From the questionnaire survey implemented within all 12 villages and based on the average human-to-dog ratio, the total rural dog population of Jiading district was estimated to be 24,058. This study generated information on the general demographics of the rural dog population in Jiading, and demonstrates an approach to the study of rural dog populations within the context of a megacity. In such a context, rural dog populations need to be considered as a critical component of animal and public health.

## Introduction

Rabies is an acute and rapidly fatal zoonotic disease caused by infection with rabies virus. The main clinical characteristics of human rabies include hydrophobia, anemophobia, and progressive paralysis (1, 2). Rabies ranks as the greatest cause of mortality amongst all Class B infectious diseases in China (under The Infectious Disease Prevention and Control Acts of China) (3). The disease is transmitted via bites or scratches from rabid animals. The retrograde axoplasmic transport of rabies virus into the central nervous system inevitably results in brain lesions and death (4, 5). All mammalian species are susceptible to rabies. However, dogs are the main carrier host species and dog-transmitted rabies accounts for the majority of human deaths related to rabies in China (6).

Rabies is present on all continents except Antarctica, causing 59,000 human deaths worldwide annually (7). The vast majority (95%) of rabies-related human deaths occur in Asia and Africa (8), among which India and China report the highest number of cases (9). The first case of human rabies in China was described in 556 BC. Currently, the persistence of rabies continues to pose a significant risk to the public health sector in China. Between 2004 and 2007, there was a rise in the number of human rabies cases in the country, which peaked at 3,300 in 2007. However, cases have steadily declined since then (10).

In Shanghai, one of the four municipalities in China, there has been a low, sporadic yet persistent occurrence of rabies during the past decade. There were 29 rabies-related human deaths reported from 2009 to 2018. Notably, all 5 rabies mortalities recorded in 2018 were caused by contact with stray rabid dogs and negligence in seeking immediate treatment including post-exposure prophylaxis and rabies immunoglobulin (11). Hence, it is imperative to immunize and manage the stray dog population to control dog-to-human rabies transmission.

Shanghai is a highly urbanized metropolis with a permanent resident population of 24 million and hence a large number of companion dogs are present. Following the implementation of the Shanghai Municipality Regulations on Dog Management there has been a significant increase in the rabies immunization coverage among owned dogs in urban areas of Shanghai. However, in rural and suburban Shanghai, the proportion of dogs vaccinated is not as satisfactory due to a lack of awareness of dog immunization and confusion on the requirements related to pet registration and management. In addition, rabies immunization in rural villages was previously performed solely by the veterinary officers in each village. The focus of their work was livestock immunization, hence limited effort was directed to the management of companion animals: pet registration and rabies immunization were not enforced. There is also a fundamental uncertainty regarding the dog population size in rural Shanghai, with no prior demographic studies having been conducted. Nevertheless, incidents of dog bites occur on a regular basis in these areas. Hence, the rural dog population remains a subpopulation of interest for rabies control and prevention in Shanghai (12).

To effectively improve the immunization coverage of rabies among rural dogs in Shanghai, an estimate of the total rural dog population in rural Shanghai via demographic surveys is needed. There are 9 administrative districts in Shanghai that contain agricultural land. This research project was led by Shanghai Animal Disease Control Center from April to June 2020 in rural villages of Jiading district. Two methods of dog population enumeration were used and compared in the study to develop a survey protocol that can be further used to enumerate the total rural dog population in the municipality of Shanghai. The study also aimed to establish a reliable set of demographic data of the owned dog population in rural Shanghai by conducting a true census survey in selected villages of Jiading.

## Materials and Methods

Shanghai is one of the four municipalities under the direct administration of the Government of the People's Republic of China. At the county level, Shanghai is divided into 16 districts and Jiading is one of the districts located in northwestern Shanghai. Jiading covers an area of 463 km^2^. It is geographically contiguous with Kunshan city to the west and Taicang city to the north, both cities being located in the neighboring province of Jiangsu. Jiading district is therefore strategically located, connecting Shanghai and Jiangsu.

**Figure 1:**
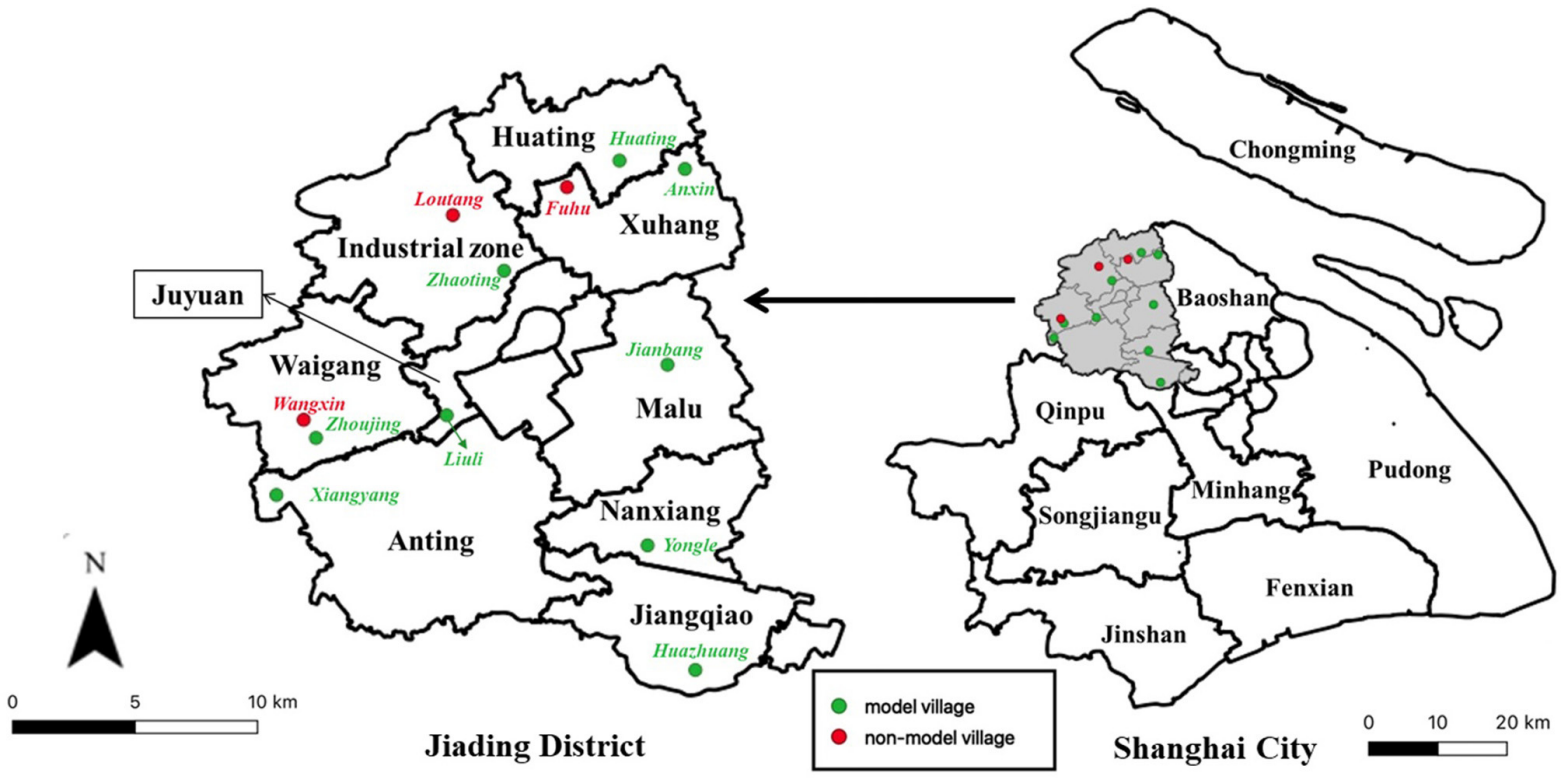
Geographical distribution of the 12 rural villages in Jiading district, Shanghai, selected for enumeration of the rural dog population. (Green markers indicate all nine model villages while red markers indicate the three selected non-model villages. A model village is one in which a set of more stringent dog management regulations was implemented by the authority).

At the township level, Jiading district is further divided into 7 towns, 2 industrial zones and 3 subdistricts (Figure 1). All towns and industrial zones contain both rural villages and urban neighborhood communities, however all 3 subdistricts have been fully urbanized and no longer contain any rural villages. Rural village and urban neighborhood communities are official terms used to describe the two types of village-level divisions under the township-level administrative divisions. Agricultural land use is only present in rural villages, not in neighborhood communities.

Rural village is an official term translated from “Nongcun” in Chinese, which is used to describe the smallest non-urban administrative division in Shanghai. Village committees are responsible for managing respective rural villages. In total, Jiading district has 129 rural villages. The rural village is the sampling unit of the study.

### Model Village Development

Responding to the municipal government's plan to eradicate rabies in Greater Shanghai, Jiading district leaders launched its very first rabies control project targeting the rural dog population. Since January 2019, 9 rural villages in Jiading have been recruited into the rabies model village development project. One rural village was selected randomly from each town or industrial zone. They were required to follow a uniform set of regulations to manage their local dog population. The regulatory policies were written by the village veterinary officers and were later enforced into practice by the local village administrative leaders. The steps taken involved a door-to-door visit and registration of all household owned dogs, authority-led mass rabies vaccination day and supplementary vaccination visits to households that were absent during mass vaccination. Rabies vaccination at these villages was subsidized fully by the local government. All vaccinated animals were given a rabies vaccination certificate. Following mandatory rabies vaccinations, regular census surveys have been conducted every quarter of the year for updated owned dog statistics.

In addition, the regulations specified assessment standards for certified rabies model villages and outlined work responsibilities of relevant village staff members. The assessment standards include: (1) 100% of all dog-owning households are informed about the rabies vaccination program, (2) 90% of all owned dog are vaccinated against rabies, (3) Reduce until elimination the number of stray dogs present in the village by capturing, desexing, and rehoming.

### Selection of Rural Villages

All 9 model villages (as defined above) were recruited into the study. In Xuhang town, Waigang town and the Jiading Industrial Zone, 3 additional non-model villages were randomly chosen for a comparative study. The study involved a comparison between 3 model villages (Anxin, Zhaoting, Zhoujing) and 3 non-model villages (Fuhu, Loutang, Wangxin). It aimed to identify the differences in the demographic profile of the local dog population under different management protocols, and the differences in dog counts obtained using two estimation methods. Two enumeration methods—a sight-resight survey and a household census survey—were implemented within 6 randomly selected villages and the estimates were compared.

In total, 9 model villages and 3 non-model villages were included in this study. In the 6 other model villages that were not included in the comparative study, sight-resight and detailed household census survey were not carried out. Instead, a simple door-to-door questionnaire was conducted to acquire the number of household owned dogs and basic household information.

### Sight-Resight as an Enumeration Method

In the 6 villages selected for the comparative study, the roaming dog population was first surveyed by a sight-resight method, following the guidelines of the World Society for the Protection Animal (13). The term *roaming dog* was used to describe both free-ranging dogs which are owned but are capable of roaming independently; and unowned, stray and homeless dogs (13–16). The counts were conducted by the village administrative staff and local veterinary officer. Recruiting local counters improved the efficiency of the counting process during the street surveys. A collective training session for the counters was held in May 2020 prior to the surveys, in order to ensure standardization of the counting methods.

Two counters surveyed the same major streets of each village using the same route, for two consecutive days from 6:00 a.m. to 8:00 a.m., on their motor vehicles. The survey aimed to cover the whole area of the village thoroughly and systematically. All roaming dogs identified in public areas (squares, roads, farming areas), including dogs that were unchained in yards without gates or fences, were recorded and photographed. The same counting processes were repeated on day 2 at the same time of the day, with the same counters and the identical route and method. Based on the information gathered on the first day, dogs that were re-sighted were noted in the counting records.

The number of dogs sighted on day 1 was tallied (*n1*). The same was done for the number of dogs sighted on day 2 (*n2*), and the number of dogs re-sighted on day 2 was determined based on records and photographs (*m2*). These values were used in the following equation to estimate the total roaming dog population ($\hat{N})\;$in the village (both seen and unseen during the survey) (17). The detection probability of the sight-resight method was calculated with m2/n1 and compared with that from other similar studies.

(1) $$\begin{array}{r} \hat{N}=\;\frac{\left(n1+1\right)\left(n2+1\right)}{m2+1}-1\; \end{array}$$

### Household Census Survey as an Enumeration Method and Demographic Investigation Tool in Model and Non-model Villages

Another enumeration method of the total roaming dog population utilized the sum of the unchained, owned dog population and the stray dog population as shown by the formula below. We obtained the unchained or unconfined, owned dog population from the household census questionnaire and the stray dog population from local officials.

$$\begin{array}{l} \text{Total Roaming Dog Population }=\\ \text{ Owned},\text{ Unchained or Unconfined Dogs }+\text{ Stray Dogs} \end{array}$$

In the 6 villages selected for the comparative study, detailed questionnaire surveys directed at dog-owning households were conducted door-to-door to gather information on the domestic dog demographics. A structured questionnaire, consisting of mainly closed-ended questions, was used to gather information on the number of dogs owned by the household. Information sought on additional demographic characteristics of the owned dogs included breed, age, use, source of acquisition, vaccination and desexing status and the owner's management strategies (including their chained/unchained status or the extent of free movements allowed inside or outside of the house). A comparison was made between information obtained from model villages and that from non-model villages. Survey staff were trained to ensure a consistent level of understanding regarding the content of the questionnaire and questionnaire delivery in a consistent manner.

The number of village stray dogs provided by the local administrative staff was based on day-to-day observation between December 2019 to June 2020, during the period of time when the study was conducted. A single person from the village administrative team was responsible for this task to minimize the possibility of double counting. Often at the time of spotting stray dogs, the staff would perform active capturing for desexing and rehoming. Hence the number of stray dogs presented in this study was the most up-to-date count indicative of the stray dog population that had not been managed. All 12 village officials provided us with the latest counts of stray dogs in their respective locale.

### Estimation of Rural Dog Population

Among all 12 villages in Jiading district that were included in the study, a concurrent tally of the rural dog population was carried out with the simple questionnaire survey implemented within each village to record the number of dog-owning households, the number of dogs owned per household, and the number of owned dogs in the village. An estimated number of stray dogs was provided by the local administrative staff. Using the human-to-dogs ratio derived from the human census data, the size of the rural dog population in Jiading district was estimated by simple extrapolation.

## Results

### Comparison of Sight-Resight and Household Questionnaire Survey as Estimation Methods in Model and Non-model Villages

A sight-resight survey and a questionnaire survey were conducted to estimate the roaming dog population in the 6 selected villages (Table 1). A paired *t*-test of the counts obtained from the two methods showed no significant difference (*t* = 1.83, *df* = 5, *P* = 0.127) between the two methods, with the counts being highly correlated (*r*_*SP*_ 0.997). The detection probability of sight-resight method in this study was calculated to be 55% (Table 1).

**Table 1:** Estimation of the number of roaming dogs in selected model and non-model villages, Jiading district, Shanghai, using sight-resight and questionnaire methods.

**Township**	**Village**	**Dog count from sight-resight**			**Estimated number of roaming dog population from sight-resight^a^ with 95% confidence interval**	**Household questionnaire survey**		**Estimated number of roaming dogs from questionnaire**
		**Day 1** ** (*n1*)**	**Day 2** ** (*n2*)**	**Resighted (*m2*)**		**Number of unchained dogs**	**Number of stray dogs**	
Xuhang	Anxin	7	5	2	15 [6, 24]	14	0	14
	Fuhu^*^	88	106	56	166 [148, 184]	131	2	133
Industrial zones	Zhaoting	9	12	4	25 [13, 37]	16	3	19
	Loutang^*^	15	12	5	34 [19, 49]	35	1	36
Waigang	Zhoujing	43	52	24	92 [75, 109]	77	0	77
	Wangxin^*^	18	16	8	35 [24, 46]	18	12	30
Total		180	203	99	367 [333, 401]	291	18	309

* *Non-model village*.

a *Estimated roaming dog population from sight-resight was calculated from the aforementioned formula in the text*.

*Detection probability = m2/n1 = 55%*.

### Comparison of Owned Dog Management Statistics and Immunization Status, Stray Dog Counts in Model, and Non-model Villages

In the 3 model villages and 3 non-model villages selected for the comparative study, a total of 1,153 questionnaires were collected from dog owners. Compared to the non-model villages, the proportion of chained or confined dogs in model villages were in general higher. In model villages Anxin, Zhaoting, and Zhoujing, the proportion of chained or confined dogs were 95.2, 93, and 79.5%, respectively. In non-model villages Fuhu, Loutang, and Wangxin, the proportion of chained or confied dogs were 65.9, 83.3, and 76% respectively. Rabies immunization rate of owned dog population in all three model villages was 100%. In non-model villages Fuhu, Loutang, and Wangxin, the immunization rate of owned dogs was 85.0, 95.7, and 97.3% respectively. While both model and non-model villages exhibited a higher than WHO recommended level (70%) of rabies immunization (18, 19), all model villages demonstrated complete rabies immunization among its owned dog population. The number of stray dogs was overall small (ranging between 0 and 3 per village), except in Wangxin where there were 12 reported stray dogs (Table 2).

**Table 2:** The number of dog-owning households, percentage of chained or confined dogs, rabies immunization rate and the number of stray and owned dogs reported in selected model and non-model villages in Jiading district, Shanghai, 2020.

**Township**	**Village**	**Number of dog-owning households**	**Number of owned dogs**	**Ratio** **(dogs/household)**	**% Chained or confined dogs**	**Rabies immunization rate among owned dogs**	**Number of stray dogs**
Xuhang	Anxin	187	290	1.55	95.2% (276/290)	100%	0
	Fuhu^*^	239	384	1.61	65.9% (253/384)	84.9% (326/384)	2
Jiading industrial zone	Zhaoting	170	227	1.34	93.0% (211/227)	100%	3
	Loutang^*^	193	209	1.08	83.3% (174/209)	95.7% (200/209)	1
Waigang	Zhoujing	332	375	1.13	79.5% (298/375)	100%	0
	Wangxin^*^	32	75	2.34	76.0% (57/75)	97.3% (73/75)	12
Total		1,153	1,560	1.35	81.4% (1,269/1,560)	95.6% (1,491/1,560)	18

* *Non-model village*.

### Demographic Profile of Owned Dogs in Model and Non-model Villages

According to the detailed household questionnaire surveys undertaken in the 6 villages, 80.4% of the 1,560 dogs registered were Chinese Garden Dogs. Most (69.1%) dogs were aged 1 to 3 years old, and owned dogs were most commonly (49.2%) homebred. Most (88.3%) of the dogs were raised for the purpose of guarding the house. Only 3.7% of the dogs were desexed (Table 3).

**Table 3:** Demographic profile of owned dogs in selected model and non-model villages in Jiading district, Shanghai, 2020.

**Village**	**Ratio^a^**	**Breed (%)**			**Age, years (%)**				**Source of acquisition (%)**			**Purpose (%)**		**Desexing rate (%)**
		**Garden**	**Hybrid**	**Pet**	**≤1**	**1–3**	**3–6**	**≥6**	**Gift from neighbors**	**Home-bred**	**External purchase**	**Guarding house**	**Companionship**	
Anxin	1.55	86.7	10.3	3.0	6.5	89.4	3.8	0.4	31.6	59.7	8.8	96.2	3.8	1.5
Fuhu^*^	1.60	88.5	9.4	2.1	5.2	78.9	12.0	3.9	22.1	74.5	3.4	97.7	2.3	1.0
Zhaoting	1.33	73.6	15.4	11.0	6.2	79.3	10.1	4.4	39.2	49.8	11.0	90.3	9.7	4.4
Loutang^*^	1.08	60.3	16.8	23.0	4.8	42.6	39.7	12.9	29.7	18.7	51.7	52.2	47.9	16.8
Zhoujing	1.12	90.1	8.8	1.1	1.3	57.3	38.1	3.2	59.7	36.0	4.3	98.1	1.9	0.5
Wangxin^*^	2.34	73.3	18.7	8.0	2.7	74.7	17.3	5.3	38.7	50.7	10.7	89.3	10.7	2.7
Total	1.35	80.4	11.5	6.4	4.4	69.1	20.4	4.4	36.7	49.2	12.4	88.3	10.0	3.7

* *Non-model village*.

a *Number of domestic dogs/Number of dog households*.

**Figure 2:**
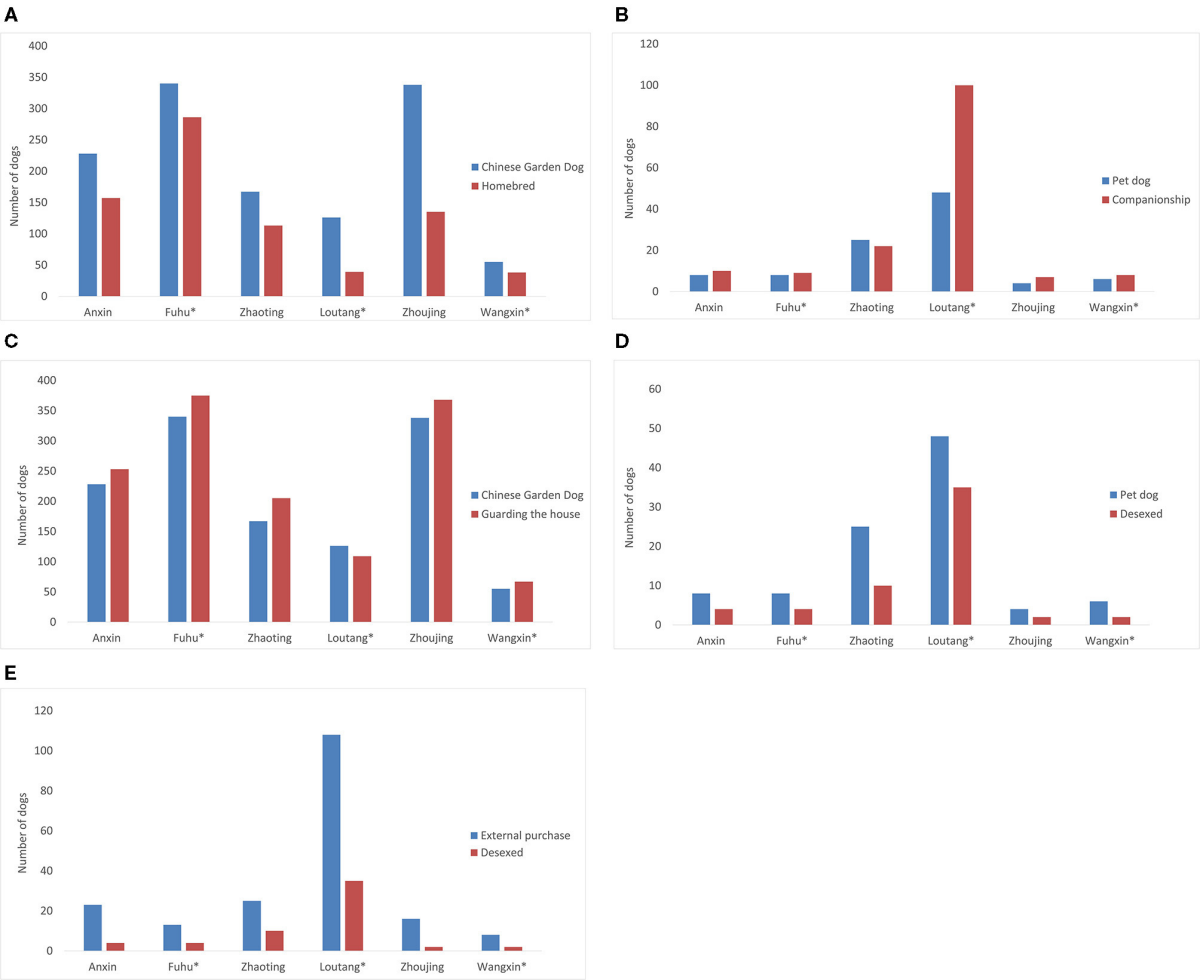
Selected pairs of demographic parameters that exhibited strong correlation in dog questionnaire surveys conducted in 6 selected villages, Jiading district, Shanghai in 2020 (*non-model villages, without rabies control): **(A)** number of Chinese Garden Dog breed versus homebred status (Spearman rank correlation *r*_*SP*_ 0.943, *P* = 0.005); **(B)** number of pet dog breeds and use for companionship purposes (*r*_*SP*_ 0.986, *P* < 0.005); **(C)** number of Chinese Garden Dog breed and use for guarding purposes (*r*_*SP*_ 1.0, *P* < 0.005); **(D)** number of pet dog breeds and being desexed *r*_*SP*_ 0.985, *P* < 0.005; **(E)** number of dogs from external purchases and being desexed (*r*_*SP*_ 0.853, *P* = 0.031).

Using Spearman's rank correlation analysis, pairs of demographic parameters that were strongly correlated were identified. The following correlations were noted: Chinese Garden Dog breed and homebred status (Spearman's correlation coefficient [rsp] 0.943, *P* = 0.005); number of pet dog breeds and being kept for companionship purposes (*r*_*SP*_ 0.986, *P* < 0.005); Chinese Garden Dog breed and use for guarding purposes (*r*_*SP*_ 1.0, *P* < 0.005); number of pet dog breeds and number desexed (*r*_*SP*_ 0.985, *P* < 0.005); and number acquired from external purchases and number desexed (*r*_*SP*_ 0.853, *P* = 0.031; Figure 2).

### The Total Number of Rural Dogs in Jiading

**Figure 3:**
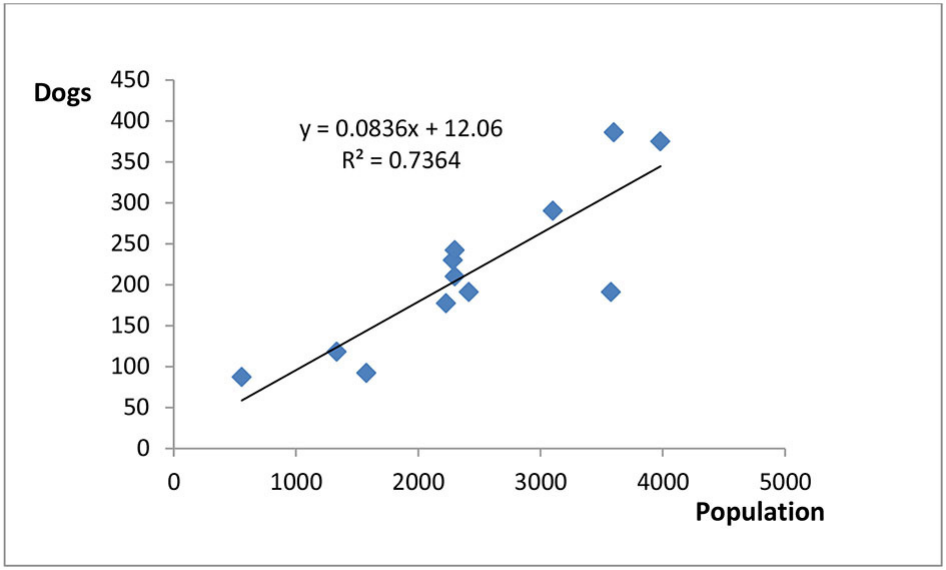
Linear regression depicting the relationship between the total dog population estimated and the total permanent resident population in the 12 selected rural villages in Jiading district, Shanghai. The human-to-dog ratio ranged from 6.4 to 18.7.

From all questionnaire surveys distributed in the 12 selected villages in Jiading, the counters obtained an estimate of the total dog population in each individual village (Table 4). Based on these results and the reported local human population size, a scatter plot displaying the relationship between the total dog population (sum of the owned dog and stray dog population) and the total permanent resident population in each village was created. The human-to-dog ratio ranged from 6.4 to 18.7, and the fitted linear regression line had an *r*^2^-value of 0.7364 (Figure 3). According to the statistical yearbook (20) of Jiading district, the permanent resident population of rural Jiading was estimated to be 287,629 as of the end of 2019. The total dog population of rural Jiading—calculated by utilizing the linear regression model constructed—was estimated to be 24,058.

**Table 4:** Collated results of dog questionnaire survey conducted in 12 selected villages, Jiading district, Shanghai.

**Township**	**Village**	**Number of households**	**Permanent population resident**	**Area (km^**2**^)**	**Number of owned dogs**	**Number of stray dogs**	**Ratio^a^**	**Dog density^b^**
Xuhang	Anxin	1,024	3,100	4.2	290	0	10.69	69.05
	Fuhu^*^	685	3,600	3.4	384	2	9.33	113.53
Jiading Industrial zone	Zhaoting	665	2,284	3.4	227	3	9.93	67.65
	Loutang^*^	911	2,300	4.3	209	1	10.95	48.84
Waigang	Zhoujing	554	3,980	3.43	375	0	10.61	109.33
	Wangxin^*^	272	5,58	3.67	75	12	6.41	23.71
Huating	Huating	793	2,300	3.7	239	3	9.50	65.41
Jiangqiao	Huazhuang	374	1,575	1	91	1	17.12	92.00
Juyuan	Liuli	550	3,576	2.5	189	2	18.72	76.40
Nanxiang	Yongle	960	2,415	3.27	187	4	12.64	58.41
Anting	Xiangyang	330	1,333	2.12	110	8	11.30	55.66
Malu	Jianbang	710	2,228	2.02	172	5	12.59	87.62
Total		7,828	29,249	37.01	2,548	41	11.30	69.95

*The number of households, permanent resident population, administrative area was accessed from the local statistical yearbook, as of the end of year 2019 (20)*.

* *Non-model village*.

a *Ratio of humans-to-dogs, Total dog population = owned dog population + stray dog population*.

b *Dog density = (owned dog population + stray dog population) per village area (sq. km)*.

## Discussion

In the selected 3 model and 3 non-model villages, we compared two different methods of estimating roaming dog population. While applying the sight-resight method based on the protocols recommended by the WSPA, it has been noted that it was relatively difficult to identify the owned, unchained dogs during the street survey as they often kept their activities within the confinement of the household. Furthermore, with the increased effort by the local committee on public education regarding specific responsibilities of pet owners, the average percentage of dogs chained or confined was found to be 81.4% (Table 2), increased significantly from the previous years (11).

Different to the sight-resight method, the roaming dog population was estimated by summing the numbers of owned, unchained or unconfined dogs reported in the household questionnaire survey and the number of stray dogs recorded by local staff. In villages with a smaller number of roaming dogs—such as Anxin and Zhaoting—the two methods yielded similar results. However, in villages with a higher number of roaming dogs—such as Fuhu and Zhoujing—the number estimated using the sight-resight method was higher than that derived from the household survey-based method (Table 1). Three underlying factors might account for this observation. Firstly, the workers recruited to execute the villages surveys are local administrative staff and veterinary officers. In villages where a smaller roaming dog population is present, the local staff are more likely to be acquainted with the individual roaming dogs and the statistics provided are likely of greater accuracy. A higher level of familiarity with the local dog population also allows for easier identification of dogs when re-encountered during the sight-resight street survey. In contrast, when there is a larger roaming population, difficulties in recognizing the features of re-sighted dogs can lead to an omission in counts. The findings may also be related to an underestimation of stray dogs by local staff from the household survey-based method. This could be due to their misunderstanding of—and a lack of a clear distinction between—owned, unchained or unconfined dogs and stray dogs. This might especially be an issue when there is a large proportion of owned but unchained or unconfined dogs in the local context. Lastly, as the local veterinary officers are involved in the management of local stray dog population, there might be a conflict of interest in reporting a high number of stray dogs, so less are reported.

The establishment of model villages in Jiading is a pioneer experiment aiming to set a standard of rabies management in rural areas of Shanghai. The study allows us to evaluate performances of such villages and decide if the same plan should be implemented at a greater scale. The model and non-model villages included in the comparative study are in proximity to each other, selected from the same town or industrial zone. Based on the results obtained from the household questionnaire survey (Table 2), there was a higher proportion of chained or confined dogs in model compared to non-model villages. The immunization coverage of rabies among owned dogs in all model villages reached 100%. In addition, the number of reported stray dogs was overall less in the model villages. These observations can be attributed to the more stringent local policy of dog population management and the focused effort in promoting responsible pet ownership in the model villages. Dog owners in model villages are discouraged from allowing their pets to roam freely in public areas and are obliged to vaccinate their dogs annually. In addition to putting in place policy and assessment guidelines, the cost of rabies vaccination is waived for all dog owners in model villages. The removal of a financial barrier could be a significant contributing factor to achieving full immunization coverage among the owned dogs. To manage the stray dogs in model villages, local technical staff are appointed to actively monitor the population dynamics of the stray dogs alongside regular mass capture. This could explain the small number of stray dogs found. However, it is noted that two of the non-model villages reported similar stray dog counts. This could indicate that stray animal management is a common work priority across all village administrative teams, model and non-model villages alike.

The results obtained from the household questionnaire surveys in the 6 villages indicated a total of 1,560 dogs owned by 1,153 households (Table 3), an average of 1.35 dogs per household. Wangxin village had the highest number of owned dogs (2.34 per household) and the highest number of stray dogs reported (Table 3). This finding can be explained by the current and ongoing land acquisition and household relocation of the 280 village residents in Wangxin. In the process of relocation, some of the owned dogs might be relocated with the household, while many others would be given to village neighbors who are staying, or these dogs might even be left behind as strays. With the rapid economic growth and urbanization of Shanghai in recent years, many agricultural villages have been demolished and incorporated into the urban landscape. As a result, many village residents have been relocated into residential compounds. This phenomenon is especially common in districts surrounding the center of Shanghai. The acquisition, reconstruction and diminution of agricultural lands has even occurred in the more peripheral Jiading district, which shares a border with neighboring Jiangsu province.

Chinese garden dog is the most popular dog breed across the 6 rural villages surveyed. These dogs are commonly homebred or acquired as a gift from neighbors for the purpose of guarding the house. There have been similar observations in all rural areas of Shanghai (11). Nevertheless, with the rapid economic development of the Greater Shanghai area, the standard of living among all rural residents has significantly improved and this has subsequently resulted in a rise in the popularity of companion dogs in recent years. Companion or pet dogs are mainly acquired from an external source; many have been desexed prior to being purchased, hence the correlation between desexing and pet dogs (Figure 2).

The main limitations of the comparative study include first of all, the small sample size. Only 6 villages participated in the detailed household census survey and sight re-sight study. A high variability of results could exist, and the power of the study could be undermined by the small sample size. The conclusion of two enumeration methods yielding no significant statistical difference may not hold true if more villages were involved in the study. In addition, although a motorized approach used in sight-resight survey was time-efficient for the staff, it might undermine the thoroughness of the search. Dogs under vehicles, in small alley ways, behind houses and trees could be missed from counting, contributing to an overall underestimation of the free roaming dog counts by the sight-resight approach. However, it is noted that the detection probability of the sight-resight method was calculated to be around 55%, close to previous studies of similar enumeration strategy performed with counters on foot (16). Therefore, the impact of the motorized approach in undercounting of dogs may not be significant.

Third, the chosen district of Jiading was the pioneer district in Shanghai that had undertaken rabies prevention and control measures; therefore, the dog population profile described in Jiading district may not be as representative and applicable to other districts. Furthermore, although the household surveys aimed to verify the number of owned dogs and their vaccination status by owner's demonstration of the registration and vaccination certificate, the results could be affected if owners intentionally conceal unregistered and unvaccinated dogs in the household. The recruitment of local village staff in the enumeration project is beneficial in this regard due to a greater level of familiarity with dogs in the vicinity and rapport built with the residents.

The most valuable aspect of this study is that the methodology incorporates a true census approach that involves door-to-door household surveys. We obtained detailed demographic, management and vaccination information on the owned dogs from household surveys in the 6 selected rural villages, and we obtained the number of household owned dogs and basic household demographic information in all 12 selected rural villages. There were no previously published studies on dog population enumeration in Shanghai and this study serves to develop a method that is statistically robust, practical and able to be readily performed at the village level and therefore widely adopted at a later date in the Greater area of Shanghai.

After testing for the difference in results obtained between the two enumeration methods, a final protocol on estimating the rural dog population in all agricultural villages in Shanghai has been established from this study. In the future, the total dog population in individual villages will be derived as the sum of the owned and stray dog population. Village-level questionnaire surveys will be organized by village administrative and veterinary staff for the purpose of evaluating the number of dog-owning households, owned and stray dogs in the locale. Although this protocol is different from the recommended sight-resight survey method, a questionnaire-based survey improves time and labor efficiency especially in the context of a relatively small dog population size in most villages and a high level of familiarity among local village staff with the local dog population. Ongoing focused sight-resight surveys can also be integrated into such a monitoring program. This study has generated dog population statistics useful for reference in the subsequent rural surveys in Shanghai.

According to the human statistics retrieved from Shanghai Statistical Yearbook (20), and the dog population estimated from all 12 villages selected in Jiading district, the estimated human-to-dog ratio was 11.3:1. The ratio was relatively higher in Huazhuang (17.1:1), Liuli (18.7:1), Yongle (12.6:1), and Jianbang (12.6:1). These villages are situated in southeast Jiading, adjacent to Minghang district. The area is in the vicinity of Shanghai's major transport interchange that incorporates the Hongqiao airport and train station. As a result, greater urbanization has occurred and only a limited amount of agricultural land is still present. Notably, the demographics of the human population in the locale consists of mainly migrant workers rather than the original village residents. This might explain a lower percentage of dog ownership. However, in Wangxin village the human-to-dog ratio was much lower (6.4:1). This might be associated with the ongoing relocation of village residents and the resultant relinquishment of dogs.

Although in this study we estimated dog population density (number of dogs per unit area of the village) in selected villages and the average dog population density from all 12 villages, it is not feasible to calculate the dog population density of the entire rural Jiading. This is because of the extensive integration between agricultural and urban land uses, which has abolished the boundaries separating rural, suburban and urban areas. The exact areas of different categories of lands, therefore, cannot be appropriately defined.

The total size of the dog population in rural Jiading was estimated to be 24,058 in this study. The total rural dog population of Shanghai could be estimated in future studies when similar enumeration projects are completed. This is an essential step prior to the rabies immunization rate being ascertained among rural dog population. At present, if a human to dog ratio of 10:1 were to be used for approximate calculations (21), the rural dog population is estimated to be around 300,000. An internal annual report from Shanghai Animal Disease Control Center has recorded that in 2019, the total number of rabies vaccinated dogs in rural areas of Shanghai was 153,771. Therefore, rabies vaccination coverage in rural Shanghai was not considered to reach the effective level of 70%. This highlights that further management effort is required to improve rabies immunization of the rural dog population and the implementation of new guidelines and regulations in rabies model village is one such initiative. In conclusion, increasing public awareness regarding rabies prevention, recruiting local support staff to implement management changes and providing free rabies vaccination services have improved local rabies immunization coverage. Evidence generated from the current study provides further strategic insights for the new edition of Regulations of Shanghai Municipality on Dog Management.

## Data Availability Statement

The original contributions presented in the study are included in the article/supplementary materials, further inquiries can be directed to the corresponding author/s.

## Ethics Statement

Ethical review and approval was not required for the animal study because activities involved are regulatory.

## Author's Note

The project is a collaboration between the University of Syndey, Shanghai Animal Disease Prevention and Control Center, and Jiading district Animal Disease Control Center.

## Author Contributions

XW and VY drafted, wrote, and edited the manuscript. ZH, SS, and JW contributed to implementing the field study methods. JL contributed to data analysis. WT, LX, and JZ contributed to tallying the results of the questionnaire surveys and sight/resight investigations. JC, GC, and YB assisted in training the survey staff and logistical aspects of the project. MW was the main supervisor of the project and editor of the manuscript. HZ was the main supervisor who managed the project and assisted in the funding aspect of the project.

## Conflict of Interest

The authors declare that the research was conducted in the absence of any commercial or financial relationships that could be construed as a potential conflict of interest.
